# Effect of nanosecond laser assisted surface modification on physical and mechanical properties of denture base materials

**DOI:** 10.1038/s41598-025-94757-w

**Published:** 2025-03-29

**Authors:** Runki Saran, Keerthi Kanneeram, V. K. Unnikrishnan, Sajan Daniel George, Aadarsh Anand Ghurye, H. Raghu Chandrashekar, Nagaraja Upadhya Perampalli, Kishore Ginjupalli

**Affiliations:** 1 Department of Dental Materials, Manipal College of Dental Sciences, Manipal Academy of Higher Education, Manipal, 576104 Karnataka India; 2 Research Associate, School of Mechanical and Aerospace Engineering, Nanyang Technological University, Singapore, Singapore; 3 Department of Atomic and Molecular Physics, Manipal Academy of Higher Education, Manipal, 576104 Karnataka India; 4 Department of Pharmaceutical Biotechnology, Manipal College of Pharmaceutical Sciences, Manipal Academy of Higher Education, Manipal, 576104 Karnataka India

**Keywords:** Laser patterning, Denture base resin, Oral health, Flexural strength, Hardness, Water sorption and solubility, Lasers, LEDs and light sources, Dental materials, Oral conditions, Prosthetic dentistry

## Abstract

Lasers are being used for modifying the surfaces of biomaterials to make them resistant to microbial adhesion. However, the effect of such surface modification on physical and mechanical properties has not been widely reported. The aim of the study was to investigate the effect of surface modification using laser patterning on the physical and mechanical properties of denture base materials. Nd: YAG nanosecond laser was used to create different patterns on two commercial denture base materials. Surface characteristics of the patterned specimens such as surface roughness, contact angle and resistance to microbial adhesion were measured. Laser patterned specimens with lower microbial adhesion were subjected to evaluation of mechanical (flexural strength and surface hardness) and physical (water sorption and solubility) properties using standard methods. Laser patterning increased the surface roughness, contact angle and the resistance to microbial adhesion. Laser patterning did not have any detrimental effects on mechanical and physical properties. However, a significant increase in surface hardness was observed in all patterned specimens. By fine-tuning the laser patterning parameters, it is possible to create surfaces with enhanced resistance to microbial adhesion without compromising the physical and mechanical properties of the material, which can ultimately lead to better oral health outcomes for denture users.

## Introduction

Laser is an acronym for Light Amplification by Stimulated Emission of Radiation that consists of a coherent monochromatic light. In dentistry, laser of a specific wavelength is focused on dental soft or hard tissues, where it is absorbed, resulting in a rapid increase in localized temperature, leading to ablation. Most commonly, diode lasers, Nd: YAG (Neodymium-doped Yttrium Aluminum Garnet) lasers, Er: YAG (Erbium-doped Yttrium Aluminum Garnet) lasers, and CO_2_ (Carbon Dioxide) lasers are used for non-invasive soft tissue procedures such as gingivectomy, frenectomy, removing excess soft tissue around teeth, etc. and for hard tissue procedures such as removal of small amount of enamel, surface conditioning, cavity preparation, etc^1,2^. Additionally, lasers have also been used widely to bring about localized surface modifications on various materials. As mentioned earlier, focusing a laser beam of sufficient energy on a small area rapidly increases the temperature leading to melting, evaporation, and/or resolidification of the material, which produces microscopic or nanoscopic structural features that alter the surface texture^3^. This phenomenon has been used to create or impart specific surface features to the material by careful selection of laser/ablation parameters such as wavelength, energy, pulse duration, repetition rate, speed of translation stage and vertical spacing between the ablation spots.

Existing literature indicates that controlled laser patterning creates surface features that significantly alters the surface characteristics of the material such as surface roughness, contact angle, surface energy, etc^4^. Laser patterning has been used to create superhydrophilic as well as superhydrophobic surfaces for a variety of applications including biomedical devices such as implants, etc^5,6^. The orientation, differentiation and proliferation of cells was found to be different on laser patterned biomaterials placed in the biological system and this has been extensively used to modify the surfaces of the implantable materials to elicit favourable biological response^7^. Surface modification of titanium alloy using laser was reported to improve its hydrophilic characteristics and create surface textures that promoted osteoblastic cell adhesion^8^. It was reported that laser assisted micro texturing of alloys, ceramics and polymers with different patterns alters their wettability, surface roughness and surface free energy characteristics with consequent effect on microbial adhesion^9^. Reduced biofilm formation was reported on laser modified titanium implant surfaces when compared to machined and grit-blasted surfaces^10^. Femtosecond laser treatment altered the surface topography of titanium alloys and decreased its wettability imparting antibacterial property, which inhibited adhesion and growth of both gram-positive and gram-negative bacteria^11^. A similar application of laser patterning on the denture base materials to reduce the microbial adhesion was recently reported^12^. While much of the research on laser modifications has concentrated on how lasers alter surface characteristics—such as roughness, wettability, and texture—there is a notable gap in understanding how these surface changes influence the other physical and mechanical properties of the materials. Additionally, existing literature indicates that lasers are widely used for surface modification of dental implant materials such as titanium and literature related to such use in denture base resins is scarce^13,14^.

As the laser surface modification involves selective removal (ablation) of the material, it leads to a significant increase in surface roughness^15^. From the structural integrity point of view, roughness on the surface may facilitate easy crack propagation and thus has a bearing on the strength of the material^16^. Similarly, a controlled laser ablation also leads to a significant increase in the surface area of the material which may have a role in influencing the water sorption and solubility^17^. With this background, the present study aims to investigate the effect of laser patterning of denture base resins on their strength, hardness, water sorption and solubility. The null hypothesis tested was that the nanosecond laser assisted surface modification does not significantly influence the bulk properties of denture base materials.

To realize this objective, two commercially available denture base resins were initially subjected to laser patterning, with the patterning parameters optimized to minimize microbial adhesion, as outlined in our previous study^12^. In the present work, a brief overview of laser patterning and its effect on surface properties is presented followed by assessment of effect of laser patterning on the mechanical and physical properties of denture base materials.

## Materials & methods

### Materials

The two commercially available heat cure acrylic-based denture base materials used in the study were Trevalon (Dentsply India Pvt. Ltd., India) and Acrypol (Ruthenium, Dental Products Pvt. Ltd., India), the details of which is given in Table 1.

**Table 1:** Details of the denture base resins used in the study.

Type of material	Brand name	Chemical composition	Manufacturer
Heat cure PMMA	Trevalon	Powder: PMMA Benzoyl peroxide Liquid: Methyl methacrylate Hydroquinone	Dentsply India Pvt Ltd, India
High impact Heat cure PMMA	Acrypol	Powder: High molecular weight PMMA Benzoyl peroxide Fibres Liquid: Methyl methacrylate Hydroquinone	Ruthenium Dental Products Pvt. Ltd., India

### Methods

#### Preparation of PMMA specimens

Compression molding technique was used to create heat-cured PMMA specimens in a way that was analogous to laboratory processing of dentures. Briefly, dental plaster was used to invest wax patterns of 65 × 10 × 2.5 mm^3^ dimensions in a denture flask. After dewaxing, a layer of separating medium (DPI heat cure Cold Mold Seal, Dental Products of India, India) was applied on the mold surfaces. The polymer powder and monomer were mixed in 2.5:1 ratio (by weight) in a ceramic jar and packed into the mold at dough stage, bench cured for 60 min and subsequently heat cured by curing at 74 °C for 8 h followed by 1 h curing at 100 °C. Post curing, the denture flask was allowed to cool to room temperature and the specimens were retrieved after deflasking. The finishing of PMMA specimens was performed using silicon carbide abrasive paper (80, 100, 220, and 400 grit sizes) fixed on to a split mandrel that was attached to a rotary handpiece operating at a constant speed of 8000 rpm. Subsequently, polishing was carried out using a slurry of Pumice and French chalk applied on buff wheels. The polished specimens were cut into 10 × 10 × 2.5 mm^3^ size using a diamond wheel. Specimens were stored in distilled water for 24 h before subjecting them to laser patterning.

#### Laser patterning

The experimental setup for laser patterning was as described in the earlier work^12^. Briefly, laser patterning was carried out using a Nd: YAG (Neodymium-doped Yttrium Aluminium Garnet) nanosecond laser (Q-Smart 450, Quantel, Lannion, France) operating at a wavelength of 532 nm with a fluence of 2.55 × 10^10^ W cm^− 2^ and pulse duration of 6 ns. The specimen was mounted on an X-Y motorized translation stage and the laser beam was focused on the specimen surface using lenses with focal length of 10 cm or 5 cm to start the process of ablation. Different patterns were created by varying the parameters such as focal length of the focusing lenses, speed of the translation stage and vertical spacing between the ablation spots, the details of which are given in Table 2. Post patterning, the specimens were sonicated in distilled water to remove any debris from their surface. Excess water was wiped off from the surface of the specimens by using tissue paper following which the specimens were air dried.

**Table 2:** Details of the patterning parameters used in the study.

Pattern	Focal length	Speed of translation stage (µm/s)	Vertical spacing between spots (µm)
1	10 cm	600	100
2		600	80
3	5 cm	600	100
4		600	80
5		400	80

#### Surface roughness measurement

Surface roughness of specimens was measured using a profilometer (Form Taly Surf 50, Taylor Hobson, United Kingdom) with a diamond stylus having 2 μm tip radius^12^. Briefly, the stylus was moved at 0.25 mm/s speed, through a distance of 1 mm, scanning the surface in three directions at three different locations per specimen. The average roughness (Ra) was calculated and expressed in µm (*n* = 3).

#### Contact angle measurement

A contact angle goniometer (HO-IAD-CAM-01B, Holmarc) was used to analyze the contact angle of specimens with distilled water^12^. With the help of a syringe, a small drop of water (10 µL) was made to fall on the specimen surface and the image was captured and analyzed using ImageJ software to measure the contact angle (*n* = 3).

#### Microbial adhesion

The adhesion of *Candida albicans* on specimens was determined by direct microscopic counting method^12^. Briefly, the specimens measuring 10 × 10 × 2.5 mm³ were placed in a petri dish containing *Candida albicans* suspension (0.5 McFarland standard) and incubated at 37 °C for 2 h. After incubation, the specimens were retrieved and rinsed with distilled water to remove any *Candida* cells that were not firmly attached to the surface. The adherent *Candida* cells were fixed on the specimen surface using 80% methanol for 30 s, then stained with crystal violet for 30 s. Subsequently, the specimens were observed under an optical microscope (Nikon Eclipse E200, New York, USA) at 100x magnification using an oil immersion objective lens. A total of 20 fields of view were counted per specimen, and the average number of adherent *Candida* cells was recorded and expressed as no. of cells/field of view. Field of view refers to the maximum area visible when looking through the eyepiece of a microscope. For example, if the total number of *Candida* cells were 14 in 20 fields of view, the average no. of cells/field would be calculated as 14/20 = 0.7 cells/field. The experiment was performed in triplicate (*n* = 3), and the average value was reported.

#### Flexural strength measurement

The flexural strength of specimens (measuring 65 × 10 × 2.5 mm^3^ ) was determined using a Universal testing machine (Instron 3366, UK). Laser patterning for these specimens was carried out on the entire length on a single surface. To evaluate the flexural strength, the specimens were placed on a supporting beam with a span length of 50 mm and subjected to a three-point loading at a crosshead speed of 5 mm/min until point of failure (*n* = 6).

The specimens were positioned on the supporting beam in such a way that the patterned surface is subjected to tensile loading during the testing. The following formula was used to compute flexural strength, which was represented in MPa.

$$Flexural{\text{ }}\;strength=3PL/b{d^2}$$

where P is the load at breaking, L is the span length, b is the specimen’s width, and d is the specimen’s thickness^18^.

#### Surface hardness measurement

A digital microhardness tester (MMT-X7A700, Matsuzawa Co. Ltd., Japan) was used to measure the surface hardness of specimens having dimensions of 10 × 10 × 2.5 mm^3^. A square-based pyramid-shaped diamond point indenter was used to apply a weight of 300 g to the specimen surface for 15 s^19^. Five indentations, spaced no less than one millimetre apart, were made at various locations on the surface and the average value was recorded. Hardness was obtained directly as Vickers Hardness Number (VHN). (*n* = 3)

#### Water sorption and solubility measurement

For the assessment of water sorption and solubility, PMMA specimens with dimensions of 10 × 10 × 2.5 mm^3^ were used^20^. In a desiccator filled with silica gel, each specimen was dried until a steady weight was reached (M1). The specimen’s weight was determined using a digital electronic balance (Sartorius BSA 224 S Analytical balance, Germany) with an accuracy of 0.0001 g. These specimens were then kept for the requisite amount of time (one week and two months) in distilled water at 37 °C. Following each time interval, the specimens were taken out of the container, any extra water on the surface was wiped off with tissue paper. Subsequently the weight of the specimen was recorded (M2) which represents the weight after water sorption.

After every sorption cycle, the specimens were put back in the desiccator, and they were then weighed at regular intervals until a constant weight after desiccation (M3) was established. This method allowed for the measurement of the amount of soluble material lost.

Utilizing the following formulas, the specimens’ water sorption and solubility were calculated and expressed in µg/mm^3^ (*n* = 3).

$$\begin{aligned} Water{\text{ }}\;Sorption{\text{ }} & ={\text{ }}\left( {M2 - M1} \right)/Volume{\text{ }}\,of{\text{ }}\;specimen \\ Water\;{\text{ }}Solubility{\text{ }} & ={\text{ }}\left( {M1 - M3} \right)/Volume{\text{ }}\;of\;{\text{ }}specimen \\ \end{aligned}$$

where, M1 = Initial weight, M2 = Weight after absorption, M3 = Final weight after desiccation, Volume of the specimen = (Length x Width x Thickness) mm^3^.

## Statistical analysis

The data obtained for control and laser patterned specimens were expressed as mean and standard deviation. The data were statistically analyzed using One-way ANOVA followed by Tukey post hoc test at a confidence interval of 95% [α = 0.05].

## Results

### Laser patterning

In the present study, laser patterning of the two commercially available denture base resins was carried out to create 5 different patterns by varying the patterning parameters such as focal length of the focusing lenses, speed of translation stage and vertical spacing between the ablation spots, and their resultant effect on surface properties, microbial adhesion and physical and mechanical properties of the materials was analysed.

**Fig. 1:**
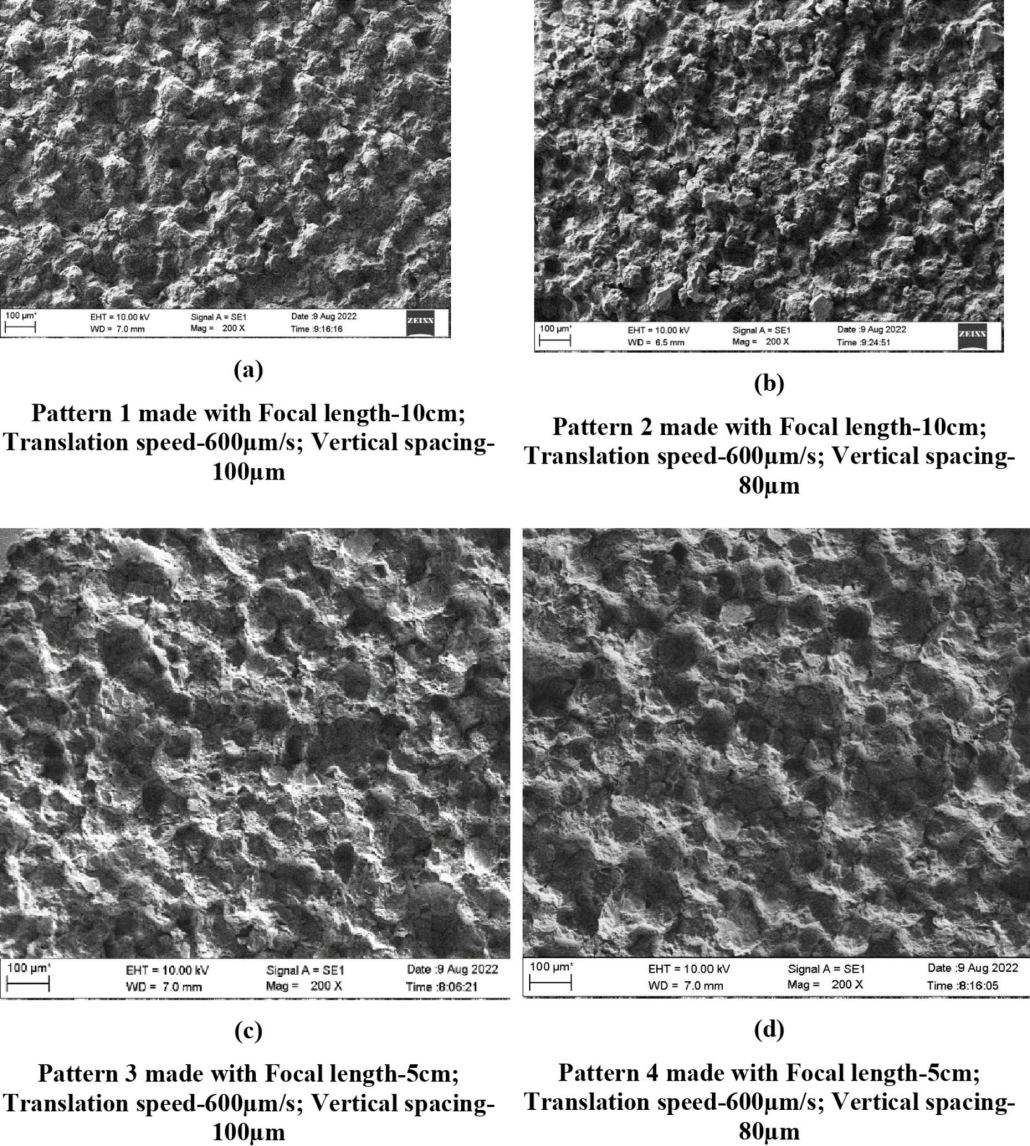

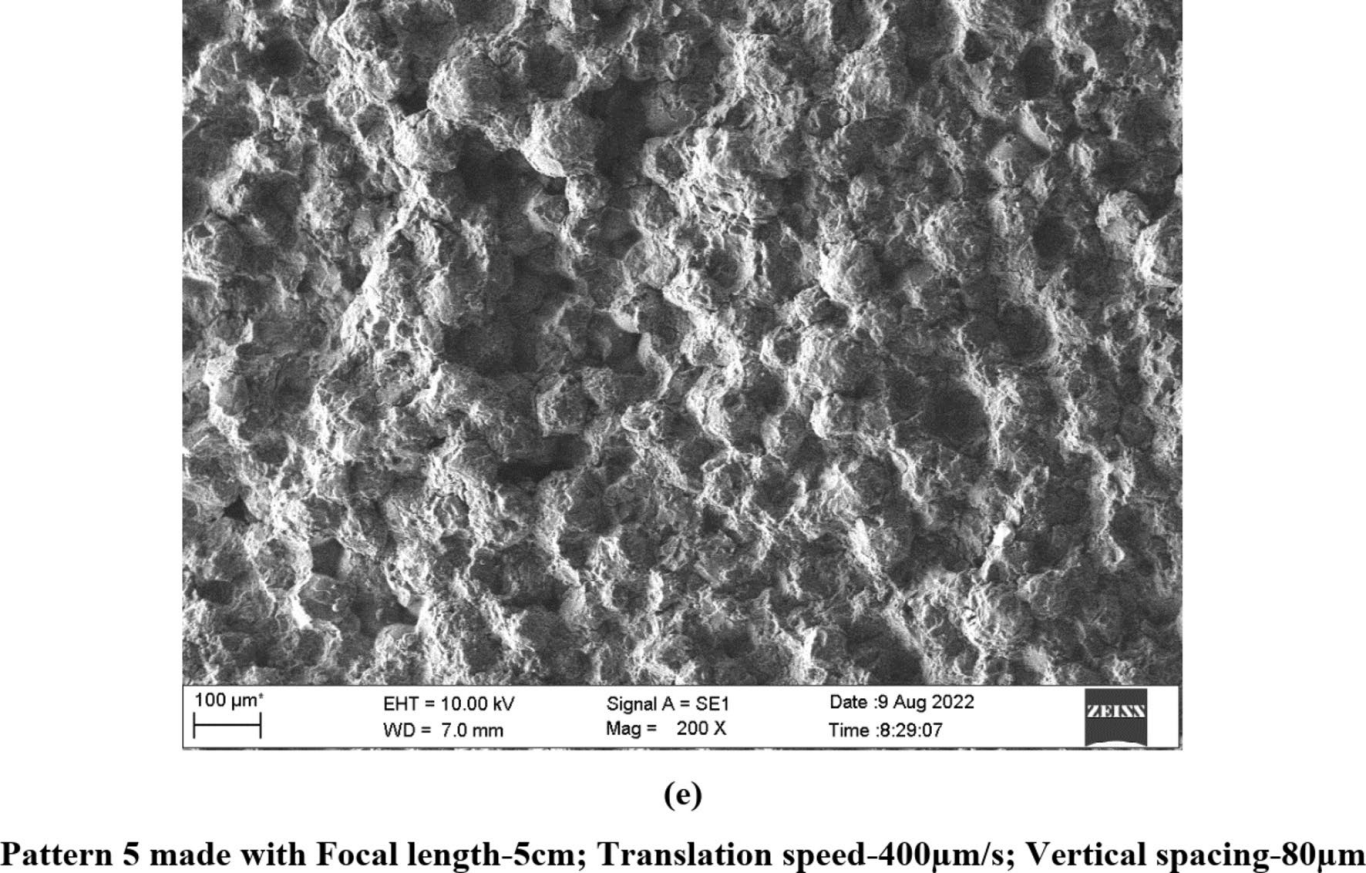
(**a**–**e**) SEM images of Trevalon Patterns made with different patterning parameters (200 × magnification).

**Fig. 2:**
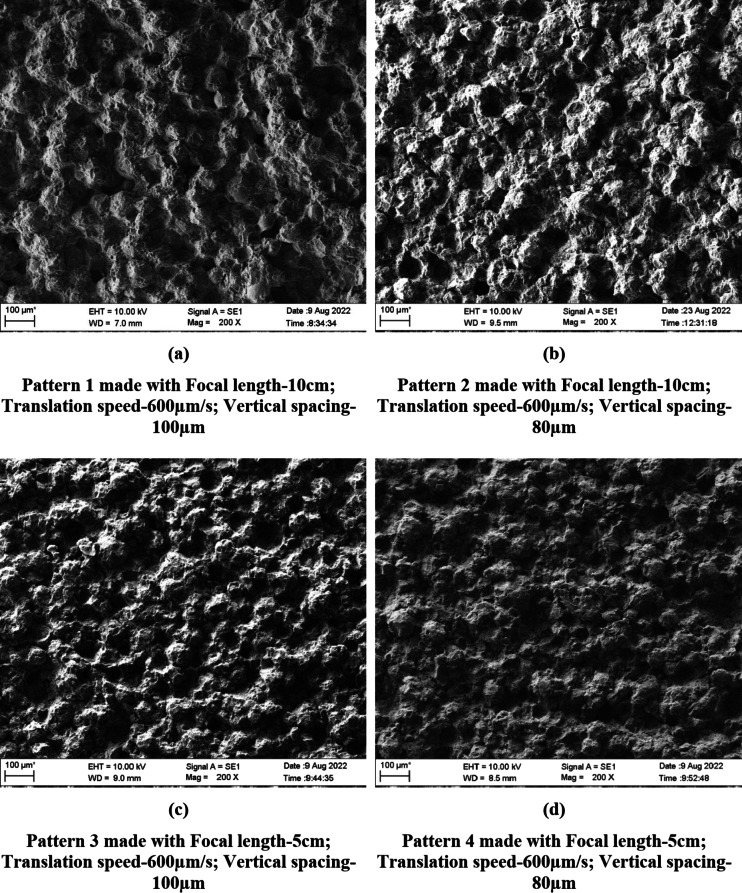

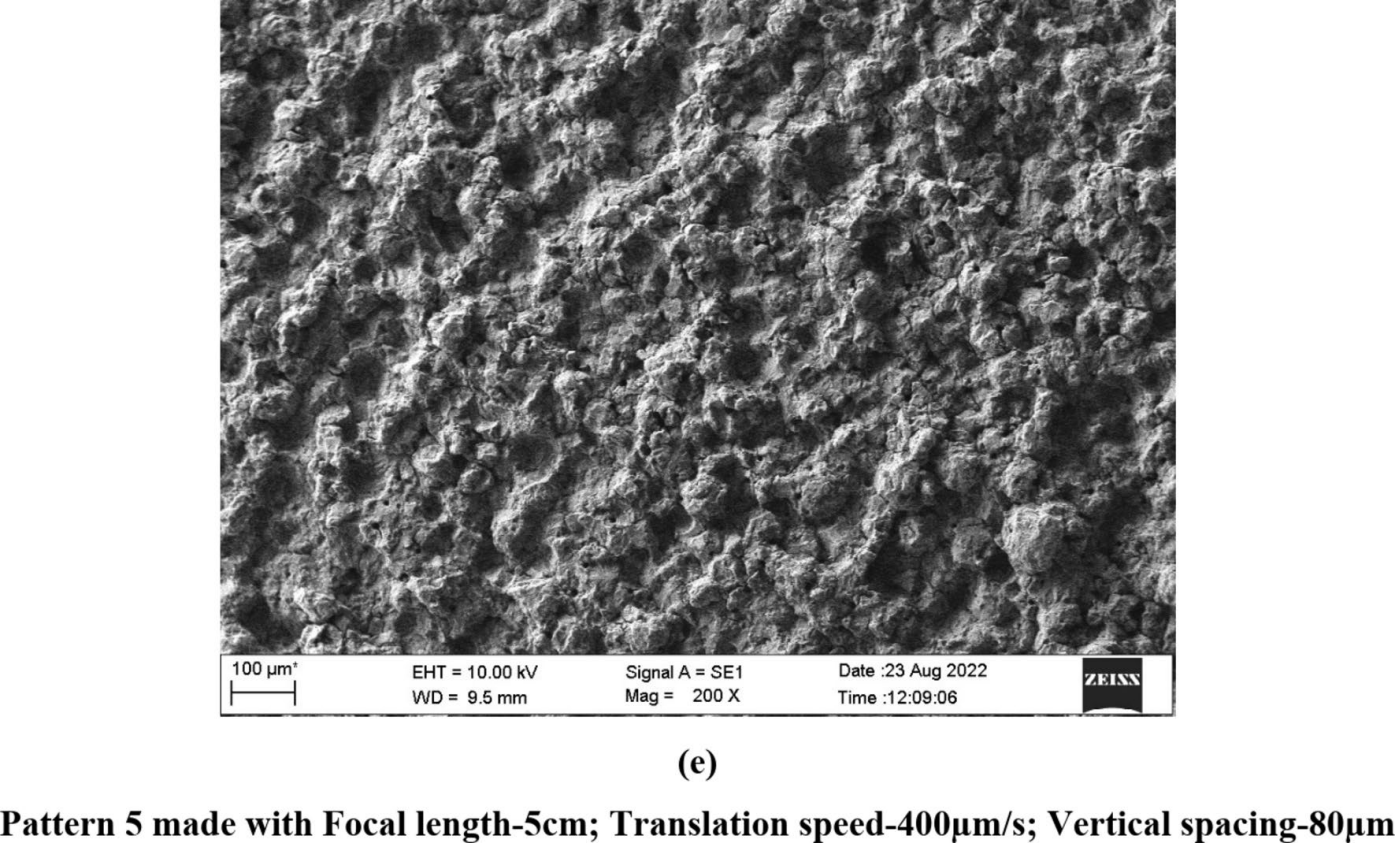
(**a**–**e**) SEM images of Acrypol Patterns made with different patterning parameters (200 × magnification).

The speed of the translation stage (600 and 400 μm/s) controlled the horizontal spacing between the ablation spots such that the spots were overlapping as evident in the SEM images (Figs. 1 and 2). Similarly, vertical spacing of 80 and 100 µm used in the study controlled the spacing between the ablation spots bringing them closer/overlapping on the vertical axis.

### Surface roughness

The surface roughness of the unpatterned (control) specimens of Trevalon and Acrypol were 0.02 ± 0.002 and 0.04 ± 0.002 μm respectively. A significantly higher surface roughness was observed for all the laser patterned specimens compared to their respective controls (Table 3).

### Contact angle

The contact angles of the unpatterned specimens of Trevalon and Acrypol were 76 ± 2° and 62 ± 2° respectively. All the laser patterned specimens showed a significantly higher contact angle compared to their respective controls (Table 3).

**Table 3:** Selected patterns with their contact angle & surface roughness.

	Contact angle (Degree) (Mean ± SD)		Surface roughness – Ra (µm) (Mean ± SD)	
	Trevalon	Acrypol	Trevalon	Acrypol
Control	76 ± 2.1	62 ± 2.1	0.02 ± 0.002	0.04 ± 0.002
Pattern 1	104 ± 1.7****	89 ± 2.2***	3.65 ± 0.4****	3.05 ± 0.6****
Pattern 2	103 ± 1.4****	95 ± 1.8****	3.85 ± 0.2****	3.38 ± 0.3****
Pattern 3	91 ± 0.72*	79 ± 1.1***	5.62 ± 0.1****	4.92 ± 0.7****
Pattern 4	96 ± 1.15**	82 ± 2.7***	5.85 ± 0.2****	5.25 ± 0.8****
Pattern 5	96 ± 1.4**	91 ± 3.4***	5.9 ± 0.1****	5.4 ± 0.3****

Asterisks (*) represent significant difference between control and laser patterned specimens.

*p < 0.05, *p < 0.01, ***p < 0.001, ****p < 0.0001.

### Microbial adhesion

**Fig. 3:**
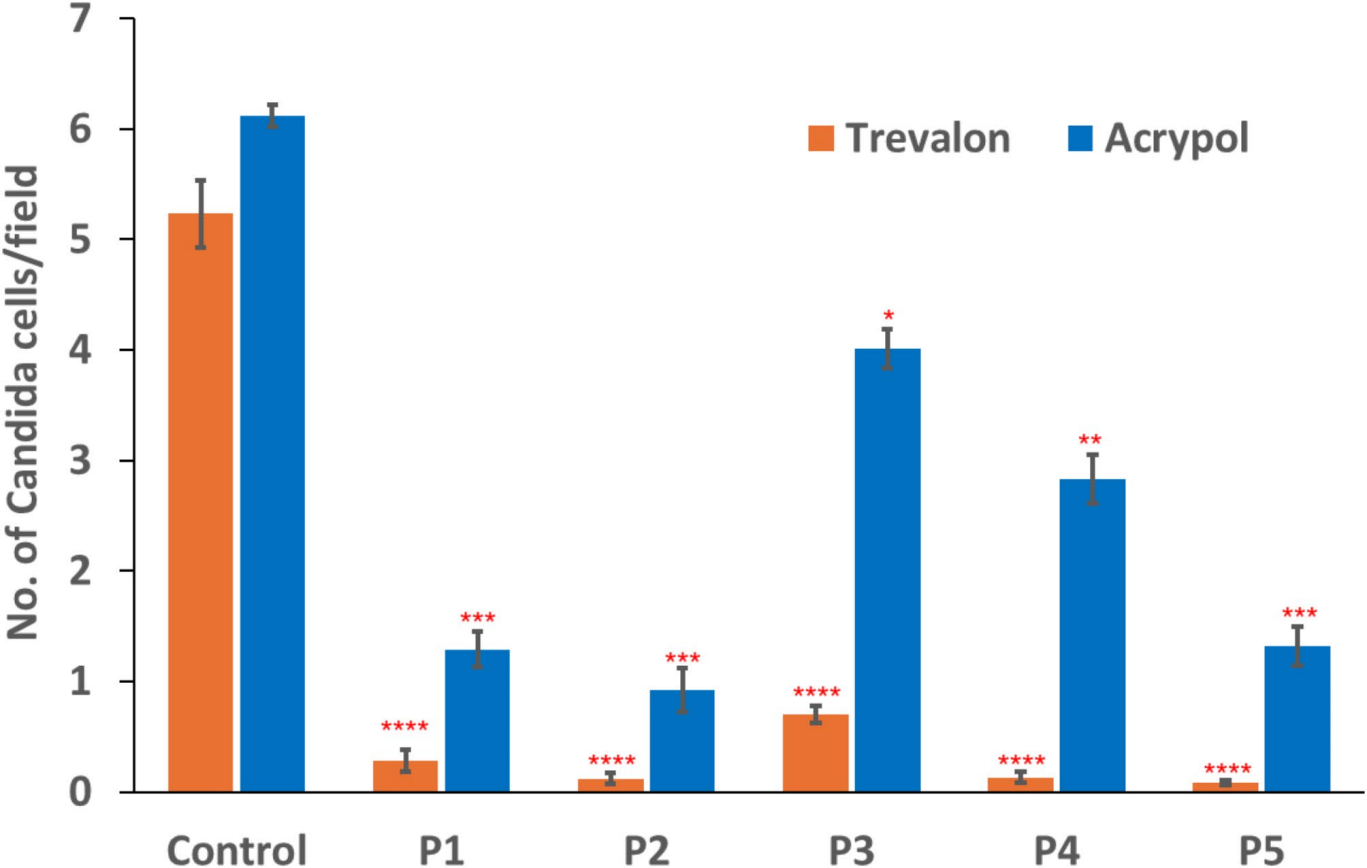
No. of Candida cells (Mean ± SD) on control & laser patterned specimens of Trevalon & Acrypol. Asterisks (*) represent significant difference between control and laser patterned specimens. *p < 0.05, **p < 0.01, ***p < 0.001, ****p < 0.0001.

The adhesion of *Candida albicans* on patterned and unpatterned (control) specimens is presented in Fig. 3. The control specimens of Trevalon and Acrypol showed 5 ± 0.3 and 6 ± 0.3 number of *Candida* cells/field respectively. All the laser patterned specimens of both the materials used in the study exhibited significantly lower number of *Candida* cells/field compared to their respective control as shown in Fig. 3.

### Scanning electron microscopy

The laser patterning process completely altered the surface topography of the denture base materials as is evident from the SEM images of PMMA specimens (Figs. 1 and 2). The ablated spots and the resolidified areas that are formed when the ablated material cools and re-solidifies can be clearly seen on the patterned surface.

All the findings appeared to be consistent for both denture base materials investigated in the present study, except for minor differences in spot size and the structure of the resolidified regions on the material surface. These variations are likely attributed to the slight differences in the composition of the two materials.

### Flexural strength

The flexural strength of control and laser patterned specimens of the two denture base materials are shown in Table 4. The unpatterned (control) specimens of Trevalon and Acrypol displayed flexural strength of 92.59 ± 3.47 MPa and 94.81 ± 3.37 MPa, respectively. For Trevalon specimens, the flexural strength of laser patterned samples was comparable to that of control specimens, with the exception of Pattern 2, which exhibited a lower strength than the control (*p* < 0.05). Similarly, no statistically significant differences in flexural strength were observed among the laser patterned Acrypol specimens compared to the control.

### Surface hardness

The surface hardness of the control and laser patterned specimens of the two denture base materials are shown in Table 4. A significantly higher surface hardness was exhibited by all the laser patterned specimens compared to their control counterparts.

**Table 4:** Flexural strength and surface hardness of 5 patterns made with Trevalon & acrypol denture base material.

	Flexural strength (MPa) (Mean ± SD)		Surface hardness (VHN) –MPa (Mean ± SD)	
	Trevalon	Acrypol	Trevalon	Acrypol
Control	92.59 ± 3.47	94.81 ± 3.37	18.7 ± 0.4	19.4 ± 1.3
Pattern 1	87.62 ± 3.68	90.72 ± 4.81	34.1 ± 1.28**	34.8 ± 1.8**
Pattern 2	86.20 ± 2.44*	89.51 ± 4.23	33.8 ± 0.9**	28.4 ± 2.4*
Pattern 3	92.38 ± 4.04	90.56 ± 4.67	39.2 ± 1.6***	31.2 ± 2.1*
Pattern 4	93.48 ± 3.02	94.04 ± 4.94	46.6 ± 1.8***	32.3 ± 1.1**
Pattern 5	93.53 ± 3.00	94.15 ± 4.41	48.2 ± 1.5***	35.2 ± 0.9**

Asterisks (*) represent significant difference between control and laser patterned specimens. *p < 0.05, **p < 0.01, ***p < 0.001.

### Water sorption

The water sorption of control and laser patterned specimens of the two denture base materials at 1 week and 2 months interval are shown in Table 5.

The water sorption at 1 week of the unpatterned (control) specimens of Trevalon and Acrypol was 14.12 ± 0.52 µg/mm^3^ and 17.15 ± 0.24 µg/mm^3^ respectively. The water sorption (at 1 week and 2 months) of laser patterned specimens of both the denture base materials did not differ significantly from their respective control specimens.

**Table 5:** Water sorption of Trevalon & acrypol specimens.

	1 week water sorption (µg/mm^3^) (Mean ± SD)		2 months water sorption(µg/mm^3^) (Mean ± SD)	
	Trevalon	Acrypol	Trevalon	Acrypol
Control	14.12 ± 0.52	17.15 ± 0.24	23.60 ± 0.12	21.34 ± 0.21
Pattern 1	14.28 ± 0.14	17.03 ± 0.82	23.18 ± 0.33	22.04 ± 0.34
Pattern 2	14.65 ± 0.38	16.90 ± 0.46	22.20 ± 0.68	20.95 ± 0.50
Pattern 3	15.08 ± 0.54	18.05 ± 0.13	24.14 ± 0.46	22.15 ± 0.66
Pattern 4	14.10 ± 0.22	18.35 ± 0.55	23.78 ± 0.15	21.01 ± 0.36
Pattern 5	13.92 ± 0.76	16.54 ± 0.74	22.90 ± 0.44	21.50 ± 0.85

### Water solubility

The water solubility of the control and laser patterned specimens of the two denture base materials at 1 week and 2 months interval are shown in Table 6.

**Table 6:** Water solubility of Trevalon & acrypol specimens.

	1 week water solubility (µg/mm^3^) (Mean ± SD)		2 months water solubility (µg/mm^3^) (Mean ± SD)	
	Trevalon	Acrypol	Trevalon	Acrypol
Control	0.4983 ± 0.0024	0.4976 ± 0.0016	1.4748 ± 0.0031	0.9917 ± 0.0036
Pattern 1	0.4967 ± 0.0012	0.4983 ± 0.0022	0.9935 ± 0.0018*	1.1933 ± 0.0018
Pattern 2	0.4961 ± 0.0031	0.4976 ± 0.0056	1.4841 ± 0.0051	0.9966 ± 0.0032
Pattern 3	0.4977 ± 0.0025	0.4975 ± 0.0024	1.4882 ± 0.0024	0.9955 ± 0.0014
Pattern 4	0.4916 ± 0.0017	0.4969 ± 0.0038	1.4929 ± 0.0062	0.9906 ± 0.0060
Pattern 5	0.4947 ± 0.0052	0.4959 ± 0.0015	0.9966 ± 0.0080*	0.9938 ± 0.0035

Asterisks (*) represent significant difference between control and laser patterned specimens. *p < 0.05, **p < 0.01, ***p < 0.001.

The water solubility at 1 week for the unpatterned (control) specimens of Trevalon and Acrypol was 0.4983 ± 0.0024 µg/mm^3^ and 0.4976 ± 0.0016 µg/mm^3^ respectively. Water solubility of all specimens made with Trevalon and Acrypol increased with time. At the end of 2 months, water solubility of all specimens was higher than their respective solubility at 1 week. For both materials, no significant difference in water solubility at 1 week was observed between the laser patterned and control specimens. However, for Trevalon, lower water solubility at 2 months was observed with Patterns 1 & 5.

## Discussion

The main aim of the study was to investigate the effect of laser patterning on the physical and mechanical properties of denture base materials. The results of the flexural strength, surface hardness, water sorption and solubility measurements of these patterned specimens and their controls (unpatterned specimens) indicate that laser patterning did not significantly alter the physical and mechanical properties of the materials and hence null hypothesis is not rejected.

In the present study, laser patterning with optimized patterning parameters increased the roughness and hydrophobicity of the denture base materials which resulted in reduced microbial adhesion. When laser is focussed on a surface, the localized rise in temperature results in melting, vaporization, and/or resolidification of the material surface. During this process, elevations and depressions are created on the surface which alter the surface topography and increases the roughness. Air pockets form between these topographical features, and prevent the liquid from spreading, thus resulting in a higher contact angle^21^. On the surfaces with high roughness, the spacing between elevations is lesser which significantly reduces the area available for microbial contact. Additionally, the air pockets between the structural features of the patterned surfaces further hinder the attachment of *Candida* cells to the denture material^22^. The findings of the present study are based on the direct counting with crystal violet staining with limitation in terms of distinguishing between live and dead candidal cells. Although, thorough washing effectively detaches the non-viable cells from the surfaces leaving the viable and adhered candidal cells for counting, additional techniques that differentiate viable and non-viable candidal cells are warranted. Such techniques not only differentiate viable and non-viable cells but also provide additional details on the likely effect of patterning morphology on the ability of the microorganisms to adhere and colonize.

Although laser patterning of the denture base materials creates different topographical changes, thus increasing their surface roughness significantly, such topographical changes are due to selective evaporation/ablation of the material. It is assumed that while laser patterning increases the surface roughness, this enhanced roughness should not be regarded as a flaw. This is because the laser patterning leads to complete melting/evaporation and resolidification of the material leaving a surface without much of surface defects. It is also likely that due to the localized temperature rise during the laser patterning, the polymer will melt and flow into the flaws or asperities. This means that the process of fracture of the material through crack initiation and propagation follows the normal course as seen with control samples. In addition, as the localized melting and solidification of denture base material during laser patterning occurs at a rapid scale, it is likely that the polymer undergoes densification leading to superior surface hardness. This could also explain why laser patterning did not negatively impact the strength. The flexural strength of a material reflects its capacity to withstand fracture under complex forces, such as those experienced from masticatory forces in the oral cavity^23^. For denture base materials, high flexural strength is crucial to ensure durability and a long service life. According to ANSI/ADA Specification No. 12 for denture base polymers, the flexural strength of denture base resins must be at least 65 MPa for clinical acceptance. In our study, all laser patterned specimens of both Trevalon and Acrypol denture base materials exceeded this minimum requirement.

Laser patterning positively influenced the surface hardness of both Trevalon and Acrypol materials, as all laser patterned specimens showed increased hardness compared to their control counterparts. As described earlier, the densification of the material due to rapid cooling of the surface during laser patterning process, could be responsible for the increased surface hardness. Similar effects have been observed in other materials, such as titanium and its alloys^24,25^. A material’s resistance to abrasion can be inferred from its surface hardness. A denture should have adequate surface hardness to resist the abrasive forces encountered during its clinical use or manual cleansing operations etc^26^.

According to ANSI/ADA specification no.12, the water sorption of denture base resins at any time should be less than 32 µg/mm^3^ for clinical acceptance. If the water sorption is higher, it may compromise the dimensional stability of the material and affect the clinical fit of the prosthesis. The results of the current investigation indicate that laser patterning has no negative effects on the water sorption of Trevalon or Acrypol denture base materials, since both showed significantly lower water sorption at all time intervals than the maximum permissible value. Water sorption in PMMA denture bases occurs when exposed to saliva, cleansing/disinfecting solutions etc., and is driven by the polarity of PMMA molecules which contain carbonyl groups that attract water molecules^27^. Patterning of PMMA specimens using optimized patterning parameters with energy levels close to the material’s ablation threshold prevents any alteration in surface chemistry, and the densification of the surface that may have occurred due to rapid resolidification of the surface, likely explains the comparable water sorption properties observed between the control and laser patterned specimens.

PMMA resin’s solubility is largely due to the leaching of unreacted monomers, plasticizers, and water-soluble components present in the resin. This solubility plays a significant role in the biocompatibility of denture base materials, as the release of these substances can elevate the risk of cytotoxic effects. Apart from this, other properties of the material such as strength, aesthetics may also be affected^28,29^. Most of the laser patterned specimens in the present study exhibited water solubility values that were similar to those of the control specimens. The fact that the patterned surface’s molecular structure and chemical composition were unaffected by the laser patterning could account for the observed phenomenon. According to ANSI/ADA specification no.12, the water solubility of denture base resins at any time should not be more than 0.04 mg/mm^3^ for clinical acceptance. In our study, both Trevalon and Acrypol denture base materials exhibited much lower water solubility at all time intervals than the maximum value specified by ADA even after patterning.

To summarize, Nd: YAG nanosecond laser was used with varying patterning parameters for surface modification of PMMA denture base resins. Laser patterning increased the surface roughness as well as the contact angle of the denture base materials. The microbial adhesion studies clearly demonstrated reduced microbial adhesion on laser patterned surfaces having higher hydrophobicity and roughness. Importantly, the laser patterning did not negatively affect the physical and mechanical properties of the materials, such as flexural strength, water sorption, and solubility. Additionally, the increase in surface hardness following laser patterning could enhance the clinical performance of the materials by making them more resistant to abrasion.

Notwithstanding these encouraging results, the present study demonstrates the ability of laser patterning in reducing the microbial adhesion on flat surfaces alone and hence requires significant efforts to induce such surface topographical changes on to irregular and curved surfaces of prostheses that significantly vary in size and shape and are patient specific. In this regard, a 6-axis translation stage that works with the help of interconnected actuators, each working independently with precise control on the position and orientation independently in the three-dimensional space can be utilized.

## Conclusions

The results of the present study demonstrate the suitability of laser patterning for optimal modification of surface features of denture base resins to enhance resistance to microbial adhesion. In addition, such modification is localized and is limited to surface of the material, without affecting the mechanical and physical properties of the material. As a result, this will create new avenues for laser patterning on dentures, making them suitable for routine clinical applications.

## Data Availability

All data that support the findings of this study are included within the article.
